# Right anterior mini thoracotomy for redo cardiac surgery: case series from North America and Europe

**DOI:** 10.3389/fcvm.2024.1427930

**Published:** 2024-06-18

**Authors:** Ali Fatehi Hassanabad, Justyna Fercho, Mortaza Fatehi Hassanabad, Melissa King, Morgan Sosniuk, Dominique de Waard, Corey Adams, William D. T. Kent, Wojtek Karolak

**Affiliations:** ^1^ Section of Cardiac Surgery, Department of Cardiac Sciences, Libin Cardiovascular Institute, University of Calgary, Calgary, AB, Canada; ^2^ Department of Cardiac Surgery, Medical University of Gdansk, Gdansk, Poland; ^3^ Department of General Internal Medicine, Faculty of Medicine, University of Calgary, Calgary, NS, Canada; ^4^ Division of Cardiac Surgery, Nova Scotia Health Authority, Dalhousie University, Halifax, NS, Canada

**Keywords:** right anterior mini thoracotomy, aortic valve replacement, redo-surgery, minimally-invasive valve surgery, minimally-invasive surgery

## Abstract

**Background:**

Right anterior mini thoracotomy (RAMT) for aortic valve replacement (AVR) is a minimally invasive procedure that avoids sternotomy. Herein, we report the outcomes of patients who underwent redo-cardiac via a RAMT approach for AVR.

**Methods:**

This case series reports the clinical outcomes of 14 consecutive redo operations, done in Calgary (Canada) and Gdansk (Poland) between 2020 and 2023. Primary outcomes were 30-day mortality and disabling stroke. Secondary outcomes included surgical times, hemodynamics, permanent pacemaker implantation (PPM), length of ICU and hospital stay, new post-operative atrial fibrillation (POAF), post-operative blood transfusion, incidence of acute respiratory distress syndrome (ARDS), rate of continuous renal replacement therapy (CRRT) and/or dialysis, and chest tube output in the first 12-hours after surgery.

**Results:**

Nine patients were male, and the mean age was 64.36 years. There were no deaths, while one patient had a disabling stroke postoperatively. Mean cardiopulmonary bypass and cross clamp-times were 136 min and 90 min, respectively. Three patients needed a PPM, 3 patients needed blood transfusions, and 2 developed new onset POAF. Median lengths of ICU and hospital stays were 2 and 12 days, respectively. There was no incidence of paravalvular leak greater than trace and the average transvalvular mean gradient was 12.23 mmHg.

**Conclusion:**

The number of patients requiring redo-AVR is increasing. Redo-sternotomy may not be feasible for many patients. This study suggests that the RAMT approach is a safe alternative to redo-sternotomy for patients that require an AVR.

## Introduction

Aortic valve replacement (AVR) is the gold standard treatment for severe, symptomatic aortic valve stenosis (AS). Despite an aging population, surgical and transcatheter advances have facilitated repeat interventions on dysfunctional native and prosthetic aortic valves. When considering re-intervening on a diseased prosthetic aortic valve, options include redo-surgical aortic valve replacement (SAVR) or valve-in-valve (ViV) transcatheter aortic valve replacement (TAVR). Several studies over the past 10 years have demonstrated favourable outcomes with each of these strategies (1–5). Generally, it is believed TAVR offers a minimally-invasive low risk procedure, but with limited durability, whereas redo-SAVR is associated with higher risk, but greater durability. Redo-SAVR via RAMT may represent a compromise, offering a less invasive option with greater durability.

Conventional SAVR is performed via full median sternotomy, while minimally-invasive SAVR can be done through either a hemi-sternotomy or a right anterior mini thoracotomy (RAMT). Although the current literature on redo-SAVR is mainly focused on redo-full median sternotomy or hemi-sternotomy approaches, there is a paucity of data reporting the clinical outcomes of redo-AVR, performed through a RAMT incision. When compared to conventional SAVR, RAMT has been shown to have similar clinical outcomes, less pain, and less blood transfusions (6–10). There is also evidence showing that patients undergoing RAMT can have an expedited return to their functional baseline secondary to quicker mobilization, better pain control, and no sternal precautions (11). RAMT access is well-liked by patients, as many associate full median sternotomy with increased morbidity and prolonged rehabilitation time. For these reasons, in the appropriate patient, RAMT is our preferred approach for redo-AVR.

Herein, we present the clinical outcomes of redo-AVR, performed via RAMT (redo-RAMT AVR) at two centers in North America and Europe. We show that a redo-AVR can be safely performed in appropriately selected patients through a RAMT approach. Our study provides original, real-world data on redo-RAMT AVR from two vastly different regions.

## Patients and methods

### Patient cohort

This case series involved retrospective collection of data to review the clinical outcomes of patients undergoing redo-RAMT AVR at a Canadian and a Polish center. All redo-operations were performed by 3 surgeons, who routinely perform minimally invasive valve surgery, between June 2020 and August 2023. This study was approved by the Conjoint Health Research Ethics Board at the University of Calgary and the Medical University of Gdansk underlying the Declaration of Helsinki (Ethics IDs: REB18-0042 and 062/2022, respectively).

### Study endpoints

Primary outcomes were death secondary to cardiac cause within 30-days of surgery and disabling post-operative stroke. Secondary outcomes included surgical times, permanent pacemaker implantation (PPM), length of intensive care unit (ICU) stay, length of hospital stay, new post-operative atrial fibrillation (POAF), post-operative blood transfusion, incidence of acute respiratory distress syndrome (ARDS), rate of continuous renal replacement therapy (CRRT) and/or dialysis, and chest tube output in the first 12-hours after surgery. Echocardiographic parameters indicating correct valve implantation was assessed, including incidence of paravalvular leak and residual mean transvalvular gradient.

### Preoperative and intraoperative considerations

**Figure 1:**
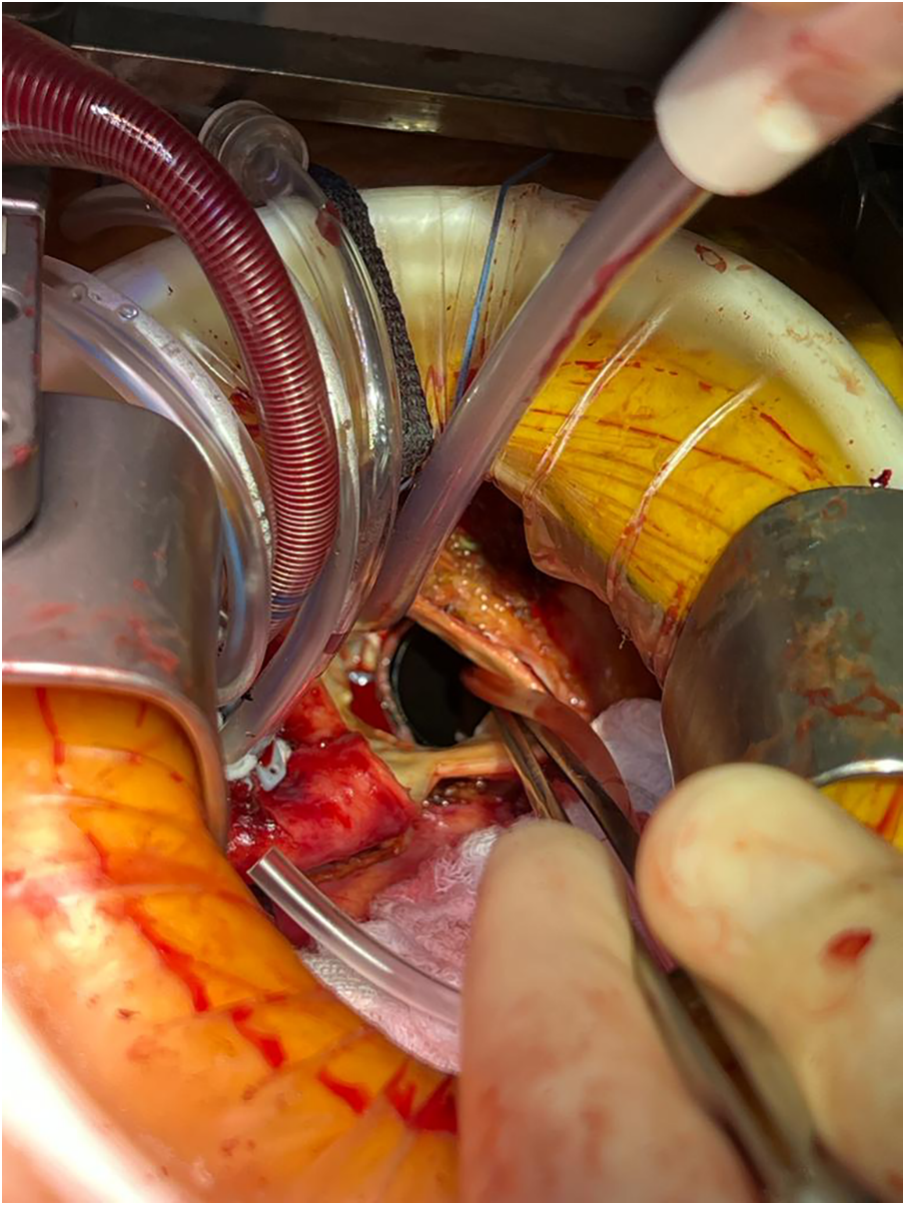
Redo-RAMT for a patient with a mechanical prosthetic aortic valve in-situ who presented with valve thrombosis. The mechanical valve was replaced with a bioprosthetic valve through a RAMT incision.

Perioperative considerations for RAMT AVR have been described in detail previously (12). The same considerations are generally applicable for redo-operations through a RAMT incision (Figure 1) and are indicated for an AVR. Briefly, the ideal candidate will not have an elevated body mass index, their aorta will not be shifted left-ward, the distance from the aortic valve to the incision is less than 9 cm, and the peripheral vessels are suitable for instituting CPB. While the authors of this study believe that a RAMT incision will provide similar exposure to the aortic valve irrespective of first-time vs. redo-surgery, since this is a complex operation, surgeons should be selective early in their experience. While no particular steps are taken in redo- vs. first-time RAMT, an important factor in selecting patients for a potential redo-RAMT AVR is the index cardiac operation. It is essential to be prepared when encountering a hostile intra-thoracic cavity with a RAMT approach as exposure, dissection, and access to the aortic valve may all be affected by dense pericardial adhesions. It may also be unsafe or unfeasible to remove a prosthetic aortic valve through a RAMT incision. In such situations, conversion to a sternotomy would be recommended.

### Inclusion and exclusion criteria

In this case series, patients were considered as possible candidates for redo-surgery via a RAMT approach if they met the anatomical requirements noted before (12). If the risk of redo-sternotomy was deemed to be too high on preoperative imaging, a stronger consideration was given for a RAMT. Furthermore, this cohort of patients were determined to have a quicker return to their functional baseline, and voiced a preference to avoid a sternotomy if it did not place them at a higher surgical risk. Patients with active infective endocarditis, previous bypass grafts, and those requiring concomitant procedures were not considered for a RAMT incision. A CT chest, abdomen, and pelvis with contrast run-off was obtained for this cohort of patients. There were no patients with missing data, so all 14 consecutive patients were included in the cohort.

## Results

### Baseline patient demographics

Fourteen consecutive patients underwent redo-cardiac surgery for an AVR through a RAMT incision. Index operations were done through sternotomy (*n* = 12), mini-sternotomy (*n* = 1), and left thoracotomy (*n* = 1, for repair of coarctation of the aorta). Nine were male and the average age of the patient cohort was 64.36 ± 11.08 years. In the cohort, 9 patients had had a previous AVR; 1 had previously undergone mitral valve replacement (MVR), tricuspid valve replacement (TVR), and aortic valve repair; 1 had undergone MVR, tricuspid valve repair, and aortic valve repair; 1 had a mechanical MVR; 1 had undergone a left thoracotomy as a child to repair coarctation of the aorta; and 1 had undergone aortic valvulotomy. Finally, the mean European System for Cardiac Operative Risk Evaluation (EuroSCORE) II was 3.77% ± 3.54% for this case series. Patient demographics are listed in Table 1.

**Table 1:** Baseline patient demographics (*n* = 14).

Variable	
Age (y)	64.36 ± 11.08
Gender (male)	9
Hypertension	12
Dyslipidemia	6
Type II diabetes	5
Renal insufficiency	4
Peripheral arterial disease	1
Chronic obstructive lung disease	5
Cerebrovascular disease	2
Prior cerebrovascular event	3
Infective endocarditis	0
AF/flutter	4
Angina	9
CCS class I	6
CCS class II	3
Presyncope	1
Syncope (at least one episode)	1
Dyspnea	14
NYHA class I	0
NYHA class II	8
NYHA class III	3
NYHA class IV	3
Indication for surgery	
Aortic stenosis	14
Index operation	
AVR	9
MVR + TVR + AVr	1
MVR + TVr + AVr	1
MVR	1
Coarct repair	1
Valvulotomy	1
Index operation (approach)	
Full median sternotomy	12
Mini-sternotomy	1
Left thoracotomy	1
EuroSCORE II	3.77% ± 3.54%

### Intraoperative details

Different types of valves were used in this case series. The type and size of the valves that were used is summarized in Table 2. A femoral cutdown was performed to establish peripheral CPB in all patients. The third rib was detached in 10 cases. There was no conversion to sternotomy and there were no concomitant procedures. The mean CPB and cross-clamp times were 137.69 ± 54.41 min and 90.47 ± 34.97 min, respectively. There was no incidence of paravalvular leak (PVL) greater than trace and the mean and peak transvalvular pressure gradients were 12.57 ± 5.94 mmHg and 25.69 ± 9.89 mmHg, respectively. Intraoperative details are summarized in Table 3.

**Table 2:** Type of valve used.

Prosthetic	
Sorin perceval	3
Medium	2
Extra-large	1
Edwards intuity (23 mm)	1
Edwards magna ease	2
23 mm	1
25 mm	1
On-X	6
21 mm	4
23 mm	1
25 mm	1
Mosaic	2
21 mm	1
27 mm	1

**Table 3:** Intraoperative details.

Variable	
Conversion to median sternotomy	0
Rib detached at costo-chondral joint	10
Peripheral cardiopulmonary bypass (cutdown on groin vessels)	14 (14)
Use of intra-operative transesophageal echocardiography	14
Del nido cardioplegia	14
Cardiopulmonary bypass time (min)	137.69 ± 54.41
Cross-clamp time (min)	90.47 ± 34.97
Paravalvular leak	
Trace or trivial	1
Mild	0
Moderate	0
Severe	0
Average residual transvalvular pressure gradient (mmHg)	
Mean	12.57 ± 5.94
Peak	25.69 ± 9.89

### Postoperative outcomes

There were no deaths at 30-days postoperatively, but 4 patients did have a neurological event postoperatively, with only 1 being disabling. The causes for the neurological events were hypoxic brain injury secondary to hypotension for 1 patient while they were undergoing continuous renal replacement therapy (CRRT); cortical laminar necrosis causing hypoxic brain injury in 1 patient; and self-limiting postoperative seizures in 2 patients. Three patients received blood products in the ICU: one patient was transfused 2 units of packed red blood cells (pRBCs), 1 patient received 4 units of pRBCs, and 1 patient received 1 unit of pRBCs. On the ward, 2 patients were transfused 2 units of pRBCs each. Three patients experienced new onset postoperative atrial fibrillation (POAF) after their operation and 3 required a permanent pacemaker (PPM). The average chest tube output in the first 12-hours after surgery was 271.43 ± 329.22 ml; of note, only one patient was taken back to the operating room emergently perioperatively for excessive bleeding. None of the patients had acute respiratory distress syndrome (ARDS). One of the patients required CRRT. Median length of ICU and hospital stays were 2 (IQR: 5) and 11 (IQR: 9) days, respectively. Postsurgical findings have been summarized in Table 4.

**Table 4:** Postoperative outcomes.

Variable	
Peri-operative mortality	0
Major disabling stroke with residual deficits	1
Emergency reoperation	1
Blood product transfusion in the ICU	
Packed red blood cells	7
Platelets	0
Average chest tube output in first 12-hours (mL)	271.43 ± 329.22 ml
Invasive ventilation (hours)	5.38 ± 2.85
Continuous renal replacement therapy	1
Hemodialysis	0
New onset atrial fibrillation	3
Permanent pacemaker	3
Dissection	0
Limb ischemia	0
Groin complications	0
Average length of stay (days)	
ICU	6.64
Hospital	14.93
Median length of stay (days)	
ICU	2
Hospital	11
Valve thrombosis	0
Valve infective endocarditis	0

## Discussion

With an aging population, repeat interventions for cardiac diseases are becoming more frequent. In most cases, the index operation is performed through a full median sternotomy. Although preoperative planning (13) and identifying patients at risk of injury during re-entry can mitigate the risk of redo sternotomy (14), it is still associated with a higher rate of complications (15, 16). Results from the multicenter European RECORD (REdo Cardiac Operation Research Database) initiative showed that conventional redo sternotomy for AVR was associated with a hospital mortality of 5.1%, major re-entry cardiovascular complications at 4.9%, and stroke at 6.6% (17). The same study found that the risk of ARDS was 10.6%; acute kidney injury (AKI) was 19.3% (where the need for CRRT was 7.2%), the need for transfusions was 66.9%, and the PPM implantation rate was 12.7% (17).

With the growth of TAVR, repeat interventions on the aortic valve are more commonly done with a ViV transcatheter approach. While there are accumulating studies that compare first time and repeat transcatheter strategies to redo-SAVR (3, 18–21), a RAMT approach should offer an important alternative for these patients for several reasons. First, the long-term outcomes of transcatheter valves is not known; second, some patients may not be suitable candidates for transcatheter approaches and transcatheter valves; third, RAMT can facilitate excellent hemodynamic results with respect to PVL and trans-valvular pressure gradients; fourth, small prosthetic aortic valves may be excised and removed through a RAMT incision when the ViV TAVR option is not feasible; and fifth, RAMT can mitigate the risks associated with proper valve deployment during TAVR, especially in patients with a prior mechanical mitral valve replacement (22).

The RAMT approach has been demonstrated to be safe for first time AVR in diverse patient populations, including octogenarians (23–26). A small number of studies have assessed the outcomes of minimally-invasive redo-AVR through hemi-sternotomy and RAMT (27–29). In a sub-population analysis of the Sutureless and Rapid-Deployment Aortic Valve Replacement International Registry (SURD-IR), Santarpino and colleagues focused on the sutureless and rapid deployment valves and reported the outcomes of 20 patients who underwent redo-RAMT AVR (27). In this registry study, among the redo-RAMT cohort, there were no deaths, while postoperative stroke rate was 4.8%, 3.6% of the patients required PPM, and bleeding requiring reoperation occurred in 8.9% of the patients (27). In a single-center study, Pindeda et al. compared the outcomes of redo-AVR via RAMT vs. median sternotomy (29). They found that in-hospital mortality was zero for the RAMT cohort vs. four (10%) in the median sternotomy group (*p* = 0.08), whereas postoperative complications occurred in six (17%) vs. 19 (46%) (*p* = 0.005) of these two groups, respectively. The median ICU and total hospital length of stay were 48-hours vs. 69-hours (*p* = 0.03), and 7-days vs. 9-days (*p* = 0.03) for the minimally-invasive and median sternotomy group, respectively (29). Although these are registry and single-center studies, respectively, they do support the safety of redo-AVR via RAMT.

The present study combines outcomes of redo-operations via a RAMT incision from a North American and a European center. We show that none of the patients died perioperatively and only one patient had a disabling stroke. Importantly, in our cohort the transfusion rate was lower that quoted in the European RECORD initiative (50% vs. 70%) (17). The same trend was noted for rate of ARDS, while similar rates were noted for CRRT in our study and the RECORD initiative. It is important to note that in this cohort, 6 of 14 patients received a mechanical prosthetic valve, highlighting the possibility of sewing in such a prosthetic through a RAMT incision in a patient with previous surgery. As expected, the transvalvular pressure gradients for these 6 patients was high, thus increasing the cohort's intraoperative valve hemodynamics. With respect to the neurological events observed in our cohort, while high (4/14 patients), their underlying cause cannot be fully attributed to intraoperative complications. Nevertheless, future studies should closely monitor and report the incidence, cause, and severity of any neurological events in patients undergoing this type of high-risk operation.

To further highlight the safety of employing a RAMT approach after prior cardiac surgery, the operations were performed by 3 different surgeons, suggesting that this strategy can be considered in carefully selected patients. These 3 surgeons routinely perform minimally invasive valve surgery, so were comfortable with a RAMT incision for redo-operations. With respect to RAMT as a first-time operation, both centers perform approximately 60 cases on an annual basis. While our cohort included patients with previous valvular operations and one patient with a previous coarct repair, none had a prior CABG surgery. Although there is a case report of a patient who underwent RAMT for redo-AVR after CABG with bilateral internal thoracic arteries (30), patent grafts can significantly increase the operative risk and these patients may be best served with a TAVR if indicated. The authors of this study believe that patent grafts and especially patent bilateral internal mammary artery grafts stand as a contraindication for redo-RAMT AVR. Nevertheless, it will be important to make note of any larger studies that report the outcomes of patients with prior CABG surgery who undergo a redo-operation through a RAMT incision. Finally, the authors would like to acknowledge that there may be concerns of encountering extensive right-sided pleural adhesions via RAMT. Surprisingly, however, very little adhesions are usually encountered through a RAMT incision even in patients whose right pleural space was opened or manipulated during their primary sternotomy.

Our study includes several limitations. First, the study size is small, which is reflective of RAMT being a relatively new approach for treating aortic valve disease. Second, the study does not report the long-term outcomes of the patient cohort. Third, the study lacks a comparator group, namely redo-sternotomy and/or redo hemi-sternotomy AVR. While comparing between surgical approaches is important, it is essential to have large sample sizes that can be propensity-matched to ensure appropriate analyses can be done when interpreting the results.

## Conclusion

With an ageing population, patients requiring redo-cardiac surgery will continue to increase. In select patients where a redo-sternotomy is not safe or feasible, a RAMT incision may be considered. Although larger studies with longer follow-up period are needed, our study suggests that RAMT can yield similar clinical outcomes to a conventional redo-sternotomy in carefully selected patients.

## Data Availability

The original contributions presented in the study are included in the article/Supplementary Material, further inquiries can be directed to the corresponding author.
